# Level of Preparedness for COVID-19 and Its Associated Factors among Frontline Healthcare Providers in South Gondar Public Hospitals, Northwest Ethiopia, 2020: A Multicenter Cross-Sectional Study

**DOI:** 10.1155/2021/6627430

**Published:** 2021-03-02

**Authors:** Ermias Sisay Chanie, Dejen Getaneh Feleke, Sheganew Fetene, Agimasie Tigabu, Sintayehu Asnakew, Tegenaw Tiruneh, Maru Mekie, Gashaw Walle Ayehu, Wubet Alebachew Bayih

**Affiliations:** ^1^Department of Pediatrics and Child Health Nursing, College of Health Sciences, Debre Tabor University, Debre Tabor, Ethiopia; ^2^Department of Emergency Medicine and Critical Care Nursing, College of Health Sciences, Debre Tabor University, Debre Tabor, Ethiopia; ^3^Department of Adult Health Nursing, College of Health Sciences, Debre Tabor University, Debre Tabor, Ethiopia; ^4^Department of Psychiatry, School of Medicine, College of Health Sciences, Debre Tabor University, Debre Tabor, Ethiopia; ^5^Department of Medical Laboratory, College of Health Sciences, Debre Tabor University, Debre Tabor, Ethiopia; ^6^Department of Midwifery, College of Health Sciences, Debre Tabor University, Debre Tabor, Ethiopia; ^7^Department of Biomedical Science (Human Anatomy), College of Health Sciences, Debre Tabor University, Debre Tabor, Ethiopia; ^8^Department of Maternal and Neonatal Health Nursing, College of Health Sciences, Debre Tabor University, Debre Tabor, Ethiopia

## Abstract

**Introduction:**

Although the efforts at global and national levels have attempted to decrease the COVID-19 pandemic, the low level of preparedness among healthcare providers is a challenge mainly in developing countries. Hence, this study is aimed at assessing the level of preparedness for COVID-19 and its associated factors among frontline healthcare providers in South Gondar public hospitals, northwest Ethiopia.

**Methods and Materials:**

A hospital-based cross-sectional study was conducted among 207 selected healthcare providers who were working in South Gondar public hospital from July 08 to August 29, 2020. A pretested structured questionnaire was used to collect data. The healthcare providers were selected through simple random sampling techniques. Both bivariable and multivariable logistic regressions with a 95% confidence interval were fitted with 95% CI to establish the associated factors with a low level of preparedness. A *p* value < 0.05 was considered statistically significant.

**Results:**

The overall level of preparedness among healthcare providers for COVID-19 was found to be 41.3% (95% CI: 37.4, 44.7). Only 81 (40.1%) healthcare providers had prepared for telling their family and friends if they are infected with COVID-19. Besides, only 23.8% of healthcare providers obtained alcohol-based hand sanitizer in every patient room. Factors associated with a low level of preparedness include being male (AOR = 2.5, 95% CI: 1.22–4.94), unmarried (AOR = 3.4, 95% CI: 1.44–8.00), and working experience less than five years (AOR = 3.4, 95% CI: 1.29-9.09).

**Conclusion:**

The level of preparedness among frontline healthcare providers towards COVID-19 was found to be very low. In the future, more emphasis should be placed on healthcare providers who are male, unmarried, and had working experience of lower than five years to decrease the burden of the COVID-19 pandemic.

## 1. Background

Coronavirus disease 2019 (COVID-19) is an emerging respiratory disease that is caused by a novel coronavirus, first detected in December 2019 in Wuhan, China [1]. On 30 January, the World Health Organization (WHO) declared that COVID-19 has been an outbreak and international concern [2]. Besides, on 04 March 2020, seventy-seven international locations had pronounced cases of COVID-19 [3].

Although early detection and prevention of onward transmission significantly reduce the burden of COVID-19 [2, 4], most African countries are much less prepared for the effective point of entry, screening, monitoring of vacationers, and treatment in the case of the contagious virus [5, 6]. According to the WHO report, the number of COVID-19 cases will be increasing in the coming era, if not improving the level of preparedness of healthcare providers [5].

Although efforts at global and national levels have attempted to improve the level of preparedness of healthcare providers worldwide to fight the COVID-19 pandemic, the burden is unacceptably high [5, 7]. The updated data on the COVID-19 pandemic are essential for healthcare providers to protect the welfare of patients and themselves [5]. However, data are limited mainly in resource-limited settings [8].

The WHO suggested that recognizing the control measures of COVID-19 like normal hand-washing, preserving physical distance, and wearing facemasks was the only choice to prevent onward transmission [8, 9]. As a result, several countries are attempting to implement preventive actions to reduce the crisis of COVID-19 [3, 9].

Globally, the healthcare providers are fallen on the COVID-19 battle [10]. On average, 7% of all cases of COVID-19 worldwide are in healthcare providers [11], around 2041 health professionals have died due to COVID-19 [12]. According to the International Council of Nurses report in Geneva, more than 450,000 nurses were infected with COVID-19 globally. Of these, 600 died [11, 13]. Moreover, data from more than 30 countries showed that death due to the COVID-19 pandemic has more than doubled in the past months among frontline nurses [11, 14].

In Ethiopia, the number of patients infected by COVID-19 has increased recently, with a very low number of healthcare providers (medical doctors, health officers, nurses, and midwives) to patient ratio. It is about 0.96/1000 in the population, and 5 times less than the minimum threshold of 4.45 per 1000 population set by the WHO [7, 15], which is a double burden for the country. Moreover, due to the mass use of public transportation, shortage of sanitation, and lack of personal protective equipment, overwhelming COVID-19 cases have occurred in the health institutions [7, 16]. Although COVID-19-infected patients are increasing in Ethiopia, the level of preparedness among frontline healthcare providers is not well investigated in the country in general and in the study area in particular. Therefore, this study is aimed at assessing the level of preparedness for COVID-19 and its associated factors among frontline healthcare providers in South Gondar public hospitals, northwest Ethiopia, to provide information to medical heroes, health institutions, policymakers, and different stakeholders to decide and to take an action.

## 2. Methodology

### 2.1. Study Area

The study was carried out in South Gondar zone public hospital, northwest Ethiopia. Debre Tabor is the capital town of the South Gondar zone, which is found 666 km far from Addis Ababa and 105 km away from Bahir Dar city. The South Gondar zone comprises one referral hospital and seven district hospitals. The hospitals serve more than 4.5 million of the residents and the nearest zonal population [17]. According to the South Gondar health office report, 429 healthcare providers work in South Gondar public hospitals (i.e., Debre Tabor referral hospital and district hospitals were 245 and 184, respectively). Moreover, the healthcare providers were working in the emergency, outpatient, and inpatient wards.

### 2.2. Study Design and Period

A hospital-based cross-sectional study was conducted from July 08 to August 29, 2020.

### 2.3. Study Population

All healthcare providers (i.e., nurses, midwives, physicians, laboratory technicians, pharmacists, psychiatric specialists, and anesthesiologists) who are working in selected South Gondar public hospitals were eligible for the study. Healthcare providers who were seriously ill and on annual leave were excluded from the study.

### 2.4. Sample Size and Sampling Technique

The sample size was calculated by considering the assumptions for single population proportion formula: the level of preparedness of healthcare providers from a previous study regarding access to the apron was 14.2% [1], *Z* = standard normal distribution value at 95% confidence level of Za/2 = 1.96, 5% of absolute precision, and 10% nonresponse rate. Hence, the total sample size was 207.

## 3. Sampling Technique and Procedure

**Figure 1:**
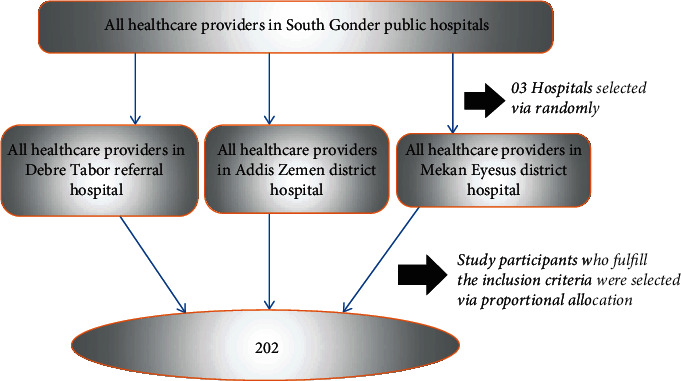
Diagrammatic presentation of the sampling procedure for the assessment of level of preparedness of frontline healthcare providers to combat the spread of COVID-19 and associated factors in South Gondar public hospitals, northwest Ethiopia, 2020 (*n* = 207).

There are a total of 8 hospitals that provide case detection, treatment, and prevention of the COVID-19 pandemic in the South Gondar zone. The hospitals were categorized into referral and district strata. Then, from the district hospital stratum, Addis Zemen and Mekan Eyesus district hospitals were selected randomly. From the referral hospital stratum, Debre Tabor referral hospital was selected since it is the only referral hospital in South Gondar zone. Moreover, the number of study participants was proportionally allocated in the selected hospitals. Then, the final study participants were selected through simple random sampling techniques by using their monthly payroll as the sampling frame in each selected hospital (Figure 1).

### 3.1. Variables

1. Dependent variables

- Level of preparedness for COVID-19 (yes/no)

(2) Independent variables

- Sociodemographic variables

- Age
- Sex
- Marital status
- Religion
- Level of education
- Profession type
- Monthly income
- Working experience
- Working units

(ii) Eligibility criteria
(iii) Exclusion criteria

Healthcare providers who were seriously ill and on annual leave were excluded.

### 3.2. Definition of Terms and Operational Definitions

Level of preparedness: based on the checklist, some of the questions elicited a “yes/no” response; besides, some elicited complete, in progress, and not started. The preparedness status was indicated by three labels: not started (≤11 of 21), in progress (<43 of 63), and complete (≥43 of 63 items). The overall level of preparedness status was indicated by two dichotomous outcomes: preparedness (≥43 of 63 items) and not preparedness (≤43 of 63 items), and then, we asked and classified the level of preparedness of the healthcare providers by using a ready score set by the WHO for COVID-19 readiness, which was classified into three levels: better prepared (over 80%), work to do (40–80%), and not ready (under 40%) [18].

### 3.3. Data Collection Tools and Procedures

The data were collected by three trained BSc nurses through a self-administered questionnaire. The questionnaire contained sociodemographic, preparedness, and readiness-related characteristics. The data collectors provided the purpose of the study for each study participant before the time of data collection.

### 3.4. Data Quality Control Method

The questionnaire was developed from the U.S. Department of Health and Human Services (HHS) Office of preparedness and response to the COVID-19 checklist [19]. One-day training and orientation were provided about the process of data collection for data collectors and supervisors. Before the actual data collection, the questionnaire was validated by pretesting on 11 eligible healthcare providers (5% of the sample size) at Ebinat District Hospital, which is one of the district hospitals in the South Gondar zone. Moreover, the filled formats were checked for completeness by the supervisor; data cleaning and double data were carried out to check for any inconsistencies, coding errors, missing values, and out of range.

### 3.5. Data Processing and Analysis

The collected data were entered into EpiData V. 4.2 and exported to STATA V.14.0 for analysis. The healthcare provider characteristics were described in terms of mean and standard deviation for continuous data. Moreover, there are percentage and frequency tables for categorical data. Both bivariable and multivariable logistic regressions with a 95% confidence interval were employed to identify factors associated with a low level of preparedness, and the variable had a *p* value less than 0.05 in the multivariable model as declared statistically significant association with a low level of psychological preparedness. Multicollinearity between the study variables was first diagnosed using the standard error and correlation matrix. Besides, Hosmer-Lemeshow statistics and Omnibus tests were performed, and Hosmer-Lemeshow's test was found to be insignificant (*p* value = 0.41), while Omnibus tests were significant (*p* ≤ 0.01) indicating the model was fitted.

## 4. Results

### 4.1. Sociodemographic Characteristics of the Respondents

Of the 207 study participants, 202 healthcare providers were included in the analysis, yielding a response rate of 97.6%. Nearly half, 104 (51.5%), of the healthcare providers were males. The mean age of the healthcare providers was 30.1 years (SD = 5.1), and 40.6% of them were between the ages of 25 and 29 years while 145 (71.8%) of the healthcare providers were married. Similarly, one hundred and forty-one (69.8%) of the healthcare providers were orthodox. Of the total healthcare providers, 91 (45.0%) were nurses. Nearly two-thirds (70.3%) of the healthcare providers had a first degree, and a majority, 163 (80.7%), of the healthcare providers had >5 years of working experience. Nearly half, 100 (49.5%), of the healthcare providers earn between 3000 and 5000 Ethiopian birr monthly. A large proportion, 127 (62.9%), of the healthcare providers were working in the ward (Table 1).

### 4.2. Level Preparedness and Readiness

The overall level of preparedness for COVID-19 among frontline healthcare providers in South Gondar public hospitals was 41.3% (95% CI: 37.4, 44.7). Among the 202 healthcare providers, 87 (43.1%) were taking training about COVID-19 infection control and updated police as required, and 123 (60.9%) had obtained complete informational materials (i.e., brochures and posters) for COVID-19. Moreover, forty-eight (23.8%) of the healthcare providers obtained an alcohol-based hand sanitizer for hand hygiene in every patient room in their institution. Similarly, 67 (33.2%) healthcare providers were getting PPE immediately outside of the patient room. Regarding the working place, 83 (41.1%) of the healthcare providers had ensured a safe working area. Besides, seventy-three (36.1%) of the healthcare providers had implemented infection prevention protocol to prevent COVID-19 transmission to their family.

A large proportion, 142 (70.3%), of the healthcare providers were ready to implement standard precautions in their working area. Besides, seventy-eight (38.6%) of healthcare providers were ready if they got COVID-19 in their working area. Nearly two-thirds, 131 (64.9%), of the healthcare providers obtained proper infection control training.

From the total of 202 healthcare providers, 78 (38.6%) of the healthcare providers were ready if they get COVID-19, whereas seventy-seven (38.1%) healthcare providers were not ready for caring for COVID-19-infected patients if their colleagues were infected with COVID-19 (Table 2). Of the total of 283 healthcare providers, 119 (58.9%), 85 (42.1%), 94 (46.5%), and 96 (47.5%) healthcare providers had a readiness to care for febrile patients, COVID-19-infected patients, overwhelmed with the new COVID-19, and COVID-19 crisis workload, respectively. Regarding support, about 91 (45.0%) and 121 (59.9%) of healthcare providers were getting support from their institution and team members, respectively. Moreover, 59 (29.2%) and 45 (22.3%) of the healthcare providers were designated points of contact for the healthcare union and family members, respectively.

**Figure 2:**
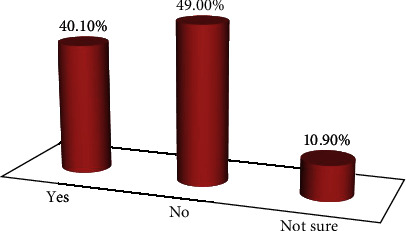
Level of preparedness of healthcare providers to tell for families and friends if they are infected with COVID-19 in South Gondar public hospitals, northwest Ethiopia, 2020 (*n* = 207).

Likewise, approximately two-thirds (59.9%) of the healthcare providers were not prepared for telling family and friends if they are infected with COVID-19 (Figure 2).

### 4.3. Factors Associated with a Low Level of Preparedness

The independent variables including age, sex, marital status, religion, level of education, profession type, monthly income, working experience, and working units were entered into the multivariable regression model. In the multivariable regression model, variables such as male, unmarried marital status, and working experience less than 5 years were found to be associated with low levels of preparedness at *p* < 0.05 with 95% CI.

The low level of preparedness among male healthcare providers was 2.5 times higher than female healthcare providers (AOR = 2.5, 95% CI: 1.22–4.94). Furthermore, the low level of preparedness among unmarried healthcare providers was 3.4 times higher than married healthcare providers (AOR = 3.4, 95% CI: 1.44–8.00). The healthcare providers who had less than five years of working experience had an increased low level of preparedness by 3.4 times than healthcare providers having working experience greater than five years (AOR = 3.4, 95% CI: 1.29–9.09) (Table 3).

## 5. Discussion

Healthcare providers in the world are at the frontline of the overwhelming COVID-19 pandemic disaster. The mortality of healthcare providers is increasing globally; 106 in Italy [10], 16 in Brazil, and 15 in Spain have died in the COVID-19 crisis [10, 11]. Moreover, countries including France, Egypt, Mexico, Pakistan, Belgium, South Korea, and Serbia have reported under 10 healthcare providers' frontline deaths [11, 13]. In general, an increased level of preparedness and readiness of health institutions is critical to saving the patients and the community at large. However, data on preparedness among healthcare providers is limited in Ethiopia for the current pandemic [8]. For this reason, the study is aimed at assessing the level of preparedness and its associated factors among frontline healthcare, which can provide data to improve the level of preparedness in a resource-limited setting, particularly in the study setting. The overall level of preparedness for COVID-19 among frontline healthcare providers was found to be 41.3%. This study revealed that healthcare providers who were males and unmarried were associated with an increased probability of a low level of preparedness for COVID-19, while healthcare providers who had greater than five years of working experience were less likely to have a low level of preparedness for COVID-19.

Our finding was lower than the study in Addis Ababa, Ethiopia, which was 52% [18], and in North Shewa Zone, Ethiopia, which was 45.6% [1]. The variation may be attributed to the difference in sample size, outcome measurement standards or instrumental measurement, and cultural differences. Moreover, this variation might be due to the difference in the study period since the healthcare provider increases their level of preparedness currently as compared to the previous era [1, 20]. On the other hand, our finding was higher than the one study conducted in vulnerable African countries including Egypt, Algeria, and South Africa [2]. This variation might be the difference in the target population, measurement criteria of the outcome variable, culture, and study period.

The healthcare providers who were male increased nearly three-folds (AOR = 2.5) for a low level of preparedness as compared with their counterparts. This finding was in line with the studies conducted in America [21]. Moreover, the healthcare providers who were unmarried increased the low level of preparedness by 3.4 times as compared with married healthcare providers. This might be because marital and romantic relationships are likely the primary source of support for many people with friends and family [22]. The healthcare providers who had less than five years of working experience increased the low level of preparedness by 3.4 times as compared to healthcare providers having working experience greater than five years. This is probably due to healthcare providers have worked in a longer time frame to have a chance of obtaining different training about infection prevention and natural disaster management, which can increase their level of preparedness and readiness for COVID-19.

## 6. Limitations of the Study

This study does have inherent limitations due to the cross-sectional nature of the study which used a snapshot of assessing the level of preparedness of healthcare providers at one point in time, which may hinder the accuracy of level preparedness and associated factors.

## 7. Conclusions

The level of preparedness among frontline healthcare providers towards COVID-19 was found to be very low. Hence, South Gondar health administrators and policymakers support the healthcare providers to meet at least the minimum standard set by the WHO, furthermore, giving more emphasis to healthcare providers who were male, unmarried, and had working experience of lower than five years to decline the COVID-19 pandemic at the healthcare setting and community level at large.

## Figures and Tables

**Table 1:** Sociodemographic characteristics of the level of preparedness among healthcare providers in South Gondar public hospitals, northwest Ethiopia, 2020.

Characteristics	Frequency (*n* = 202)	Percent (%)
Age (year)		
<25	31	15.3
25-29	82	40.6
30-34	49	24.3
>34	40	19.8
Sex		
Male	104	51.5
Female	98	48.5
Marital status		
Unmarried	50	24.8
Married	145	71.8
Others^∗^	7	3.5
Religion		
Orthodox	141	69.8
Muslim	52	25.7
Other^∗∗^	9	4.5
Level of educational status		
Diploma	50	24.8
Degree	142	70.3
Masters and above	10	5.0
Professional type		
Nurse	91	45.0
Midwifery	47	23.3
Doctor	23	11.4
Laboratory technique	14	6.9
Pharmacy	19	9.4
Others^∗∗∗^	8	4.0
Monthly income		
<3000 ETB	16	7.9
3000-5000 ETB	100	49.5
3.000-7000 ETB	67	33.2
>7000 ETB	19	9.4
Work experience		
≥5 years	163	80.7
<5 years	39	19.3
Working unit		
Ward	127	62.9
Outpatient	41	20.3
Emergency	34	16.

Others^∗^ = divorces/widowed; others^∗∗^ = protestant/catholic; others^∗∗∗^ = psychiatric specialty/anesthesia.

**Table 2:** Level preparedness and readiness for COVID-19 among frontline healthcare providers in South Gondar public hospitals, northwest Ethiopia, 2020.

Variables	Frequency	
	Completed (*N*, %)	In Progress/not started (*N*, %)
Education/training about COVID-19 infection control and update police as required?	87 (43.1)	115 (56.9)
Informational materials (e.g., brochures and posters) on COVID-19?	123 (60.9)	79 (39.1)
Alcohol-based hand sanitizer for hand hygiene is available in every patient room?	48 (23.8)	154 (76.2)
PPE available immediately outside of the patient room is provided	67 (33.2)	135 (66.8)
Ensuring safety in working place	83 (41.1)	119 (58.9)
Readiness to implement ever standard precautions	142 (70.3)	60 (19.3)
Activities to prevent COVID-19 transmission to family members	73 (36.1)	129 (63.9)
Readiness for caring for febrile patients	119 (58.9)	83 (41.1)
Readiness of self away from family members	107 (53.0)	95 (47.0)
Readiness for caring COVID-19-infected patients	85 (42.1)	117 (57.9)
Readiness overwhelmed with the new COVID-19	94 (46.5)	108 (53.5)
Readiness for telling family and friends if infected with COVID-19	81 (40.1)	121 (59.9)
Readiness for caring COVID-19-infected patients if their colleagues are infected with COVID-19	77 (38.1)	125 (61.9)
The readiness of the institution to support healthcare providers	91 (45.0)	111 (55.0)
Readiness COVID-19 crisis that increased workload	96 (47.5)	106 (52.5)
Proper infection control training has been given	131 (64.9)	71 (35.1)
Support from your team members	121 (59.9)	81 (40.1)
Redness that might eventually get COVID-19 at work	78 (38.6)	124 (61.4)
Determine a contingency staffing plan?	63 (31.2)	139 (68.8)
Designate a point of contact for the healthcare union?	59 (29.2)	143 (70.8)
Designate a point of contact for the family members?	45 (22.3)	157 (77.7)
The overall level of preparedness		
Good	89 (44.1)	
Poor	113 (55.9)	

**Table 3:** Factors associated with a low level of preparedness for COVID-19 among healthcare providers for COVID-19 in South Gondar public hospitals, northwest Ethiopia, 2020.

Variables		Low level of preparedness		OR (95% CI)		
		Yes (*n* = 119)	No (*n* = 83)	COR	AOR	*p* value
Age (year)	<25	20	11			
	25-29	44	38	0.6 (0.27-1.49)	0.7 (0.22-2.20)	0.530
	30-34	27	22	0.7 (0.26-1.70)	0.8 (0.24-2.86)	0.772
	>34	28	12	1.3 (0.47-3.48)	1.9 (0.53-7.19)	0.318
Sex	Male	74	30	2.9 (1.6-5.2)	2.5 (1.22-4.94)	0.012
	Female	45	53	1	1	—
Marital status	Unmarried	38	12	2.6 (1.3-5.5)	3.4 (1.44-8.00)	0.005
	Married	79	66	1	1	
	Others^∗^	2	5	0.3 (0-1.8)	0.2 (0.03-1.43)	0.108
Religion	Orthodox	85	56	0.4 (0.09-2.2)	0.3 (0.04-1.97)	0.20
	Muslim	27	25	0.3 (0.6-1.6)	0.3 (0.04-1.95)	0.20
	Other^∗∗^	7	2	1	1	
Level of educational status	Diploma	37	13	4.3 (1.1-17.6)	4.9 (0.81-29.0)	0.08
	Degree	78	64	1.8 (0.5-6.)	1.8 (0.33-9.84)	0.48
	Masters and specialty	4	6	1	1	
Professional type	Nurse	57	34	1	1	1
	Midwifery	26	21	0.7 (0.36-1.50)	0.6 (0.2331.42)	0.232
	Doctor	11	12	0.5 (0.22-1.37)	1.0 (0.32-3.11)	0.990
	Laboratory technique	9	5	1.1 (0.33-3.47)	1.1 (0.23-5.09)	0.925
	Pharmacy	12	7	1.0 (0.37-2.4)	0.9 (0.24-3.16)	0.830
	Others^∗∗∗^	4	4	0.6 (0.14-2.54)	0.4 (0.60-2.66)	0.343
Monthly income	<3000 ETB	10	6	1	1	
	3000-5000 ETB	63	37	1.0 (0.34-3.04)	0.8 (0.21-3.32)	0.969
	3.000-7000 ETB	36	31	0.7 (0.22-2.14)	0.4 (0.97-2.04)	0.527
	>7000 ETB	10	9	0.7 (0.17-2.58)	0.4 (0.60-2.72)	0.557
Work experience	<5 years (Ref)	31	8	3.3 (1.41-7.69)	3.4 (1.29-9.09)	0.01^∗∗^
	≥5 years)	88	75	1	1	
Working unit	Ward	75	52	1	1	
	Out patient	19	22	0.6 (0.29-1.21)	0.7 (0.31-1.67)	0.438
	Emergency	25	9	1.9 (0.83-4.46)	1.5 (0.60-4.10)	0.411

^∗^Significant at <0.05; ^∗∗^significant at <0.01; COR = crude odds ratio; AOR = adjusted odds ratio; Ref = reference category; CI = confidence interval.

## Data Availability

Data will be had upon request from the corresponding author.

## Abbreviations

AOR: Adjusted odds ratio
COR: Crude odds ratio
COVID-19: Coronavirus infectious disease 19
WHO: World Health Organization.
